# Applications of Optical Coherence Tomography in the Ocular Diagnosis: From the Tear Film to the Sclera

**DOI:** 10.3390/diagnostics12030673

**Published:** 2022-03-10

**Authors:** Claudio Iovino, Valentina Di Iorio, Raffaella Brunetti-Pierri, Michele Lanza

**Affiliations:** Eye Clinic, Multidisciplinary Department of Medical, Surgical and Dental Sciences, University of Campania ‘Luigi Vanvitelli’, Via Pansini 5, 80131 Naples, Italy; claudioiovino88@gmail.com (C.I.); valentina.diiorio@unicampania.it (V.D.I.); raffaella.brunettipierri@unicampania.it (R.B.-P.)

The latest technological developments have radically impacted the daily practice of ophthalmologists, thanks to the advent of novel diagnostic tools that facilitate an early diagnosis and allow a better management of ocular disorders.

Optical coherence tomography (OCT) has been widely used in the last decades both in research and in clinical practice to image posterior and anterior segment eye structures [1]. The first in vivo retinal images were obtained in 1993 by Swanson et al. [2], although the first time-domain OCT instruments only became commercially available in 1996.

Spectral-domain (SD)-OCT is a subset of Fourier-domain detection and is currently the standard for ophthalmic instruments with an imaging speed range from 25,000 to 130,000 A-scan per second [3]. 

The second type of Fourier-domain detection is the Swept Source (SS)-OCT with a swept laser working with longer wavelengths (1050 nm) and faster imaging speeds [3]. In particular, SS-OCT has less attenuation from ocular opacities and improved image depth [4]. OCT has many applications in the retinal field, but the development by Spaide of enhanced-depth imaging (EDI)-OCT has allowed the imaging of deeper retinal layers, including the choroid [5]. EDI-OCT and SS-OCT are particularly helpful for the analysis of a large choroidal vessel (Haller layer), medium choroidal vessel (Sattler layer), and/or the choriocapillaris layer with a clear distinction between the choroid’s outer and inner boundaries [5]. EDI-OCT has been also used to provide a detailed analysis of the sclera or to measure scleral thickness in a non-contact approach [6].

From a technical point of view, arranging successively recorded A-scans along one scanning direction in a 2D array forms a tomogram or brightness scan (B-scan).

Alternatively, the en face image is reconstructed from full 3D volumes either by direct slicing or through axial projection in post processing. In other terms, en face OCT acquires a dense volume of cross-sectional B-scan data projected onto an en face (coronal) plane [7]. The success of modern en face OCT lies in its easy image interpretation.

Originally developed to investigate the retina and the optic nerve head, OCT provides a rapid and non-invasive way to image the eye in vivo [1]. It was not until 1994 that Izatt et al. reported the first anterior segment (AS)-OCT evaluation [8]. This tool seems particularly helpful in the evaluation of the AS structures in heathy and diseased eyes [9,10] and in the clinical assessment and follow-up of patients with angle closure and other angle anomalies [11], in pre-and postsurgical evaluations [12,13]. Even changes in tear meniscus dynamics and several other parameters such as volume, tear meniscus height and turbidity could be determined with AS-OCT [14].

Optical coherence tomography angiography (OCTA) is the most relevant evolution based on OCT that enables the detailed visualization of the retinal and choroidal vasculature without using any intravenous dye [15]. OCTA technology is based on detecting the differences in amplitude, intensity or phase variance between sequential B-scans taken at the same location of the retina. It allows the direct visualization and measurement of the foveal avascular zone area and provides morphologic and quantitative vascular information on macular microcirculation, with good reproducibility and repeatability. OCTA is currently used for the diagnosis and management of many chorioretinal disorders including age-related macular degeneration, diabetic retinopathy, myopia, pachychoroid spectrum disease, neurodegenerative disorders and inflammatory and vascular retinal disorders [16,17,18,19,20].

More recently, OCTA has also been used for the examination of anterior segment ocular vasculature in healthy and diseased eyes [21,22].

This Special Issue aims to create a multidisciplinary forum of discussion about the clinical and research applications of OCT and OCTA in the ocular diagnosis of different subfields of ophthalmology.

Results of retinal and choroidal characteristics in patients with choroideremia [23], central serous chorioretinopathy [24] and erectile dysfunction [25] were presented.

The role of the OCT and OCTA in ocular surgery was also discussed.

D’Aloisio et al. investigated early retinal vascular and functional changes in patients undergoing vitreoretinal surgery for idiopathic epiretinal membrane or macular hole (MH) using a widefield swept-source OCTA [26].

Kal et al. evaluated the quantitative morphological changes in lamellar MHs based on spectral-domain OCT examinations, assessing the correlations between minimal retinal thickness, reading vision and best corrected visual acuity over a 36 month follow-up period [27].

Interestingly, Rizzo et al. demonstrated how an artificial intelligence-based analysis of preoperative OCTA images of eyes affected by a full-thickness MH could be useful to support systems in setting up visual acuity recovery prediction [28]. Particularly, the combination of preoperative superficial and deep plexuses images showed a significant morphological predictive performance for visual acuity recovery [28]. We hope that readers will appreciate this issue, which is meant to be an attempt to report the last evidence on the applications of OCT and OCTA in the diagnosis and management of ocular disorders.
